# The Unfolded Protein Response in a Murine Model of Alzheimer’s Disease: Looking for Predictors

**DOI:** 10.3390/ijms242216200

**Published:** 2023-11-11

**Authors:** Giulia Sita, Agnese Graziosi, Camilla Corrieri, Luca Ghelli, Sabrina Angelini, Pietro Cortelli, Patrizia Hrelia, Fabiana Morroni

**Affiliations:** 1 Department of Pharmacy and BioTechnology—FaBiT, Alma Mater Studiorum University of Bologna, Via Irnerio 48, 40126 Bologna, Italy; giulia.sita2@unibo.it (G.S.); agnese.graziosi2@unibo.it (A.G.); camilla.corrieri2@unibo.it (C.C.); luca.ghelli3@unibo.it (L.G.); s.angelini@unibo.it (S.A.); fabiana.morroni@unibo.it (F.M.); 2 Department of Biomedical and NeuroMotor Sciences—DiBiNeM, Alma Mater Studiorum University of Bologna, Via Altura 3, 40139 Bologna, Italy; pietro.cortelli@unibo.it

**Keywords:** Aβ oligomers, aging, Alzheimer’s disease, neurodegeneration, UPR

## Abstract

Alzheimer’s disease (AD) represents the most frequent type of dementia worldwide, and aging is the most important risk factor for the sporadic form of the pathology. The endoplasmic reticulum (ER), the main cellular actor involved in proteostasis, appears significantly compromised in AD due to the accumulation of the β-amyloid (Aβ) protein and the phosphorylated Tau protein. Increasing protein misfolding activates a specific cellular response known as Unfolded Protein Response (UPR), which orchestrates the recovery of ER function. The aim of the present study was to investigate the role of UPR in a murine model of AD induced by intracerebroventricular (i.c.v.) injection of Aβ_1–42_ oligomers at 3 or 18 months. The oligomer injection in aged animals induced memory impairment, oxidative stress, and the depletion of glutathione reserve. Furthermore, the RNA sequencing and the bioinformatic analysis performed showed the enrichment of several pathways involved in neurodegeneration and protein regulations. The analysis highlighted the significant dysregulation of the protein kinase RNA-like ER kinase (PERK), inositol-requiring protein 1α (IRE1α) and activating transcription factor 6 (ATF-6). In turn, ER stress affected the PI3K/Akt/Gsk3β and MAPK/ERK pathways, highlighting Mapkapk5 as a potential marker, whose regulation could lead to the definition of new pharmacological and neuroprotective strategies to counteract AD.

## 1. Introduction

Alzheimer’s disease (AD) is the leading cause of dementia and cognitive impairment worldwide, affecting 3–4% of adults over 65 [1]. AD is also a principal cause of disability and morbidity; thus, while recent findings are promising, the social and economic burden of this disease will continue to be huge and unsustainable. The neuropathological features characterizing AD include the extracellular accumulation of toxic species of β-amyloid (Aβ) protein, in particular Aβ_1–40_ and Aβ_1–42_ to form amyloid plaques, the deposition of intracellular neurofibrillary tangles (NFT) of hyperphosphorylated Tau protein, and neurodegeneration due to uncontrolled activation of microglia responsible for the secretion of neurotoxins and inflammatory factors [2].

Aging represents the time-dependent physiological functional decline that is also the most important risk factor for many diseases, including AD. Given the rate at which the human population is aging, it is critical to find ways to protect against or even reverse the effects of aging in order to preserve cognitive integrity. Indeed, due to the growing aging population and the increased burden of health care for AD patients, the research on this disease is rapidly expanding.

One of the most important differences between aging and AD is that the number of neurons does not change during aging, but neuron and synapse loss represent the hallmarks of AD. On the contrary, the proinflammatory and oxidative environment promoted by aging is worsened in AD, and chronic inflammation seems to be a common feature between aging and AD [3]. Indeed, AD-related pathways can be altered by oxidative stress, as p38, a member of the mitogen-activated protein kinase (MAPK) family, is activated by Aβ-mediated oxidative stress. 

Aging, inflammation and oxidative stress represent an inseparable triad, in which all of them contribute equally and feed others, creating a positive feedback loop. At the same time, cellular defense mechanisms weaken; thus, the accumulated damages cannot be repaired efficiently, leading to loss of function and finally cell death [4]. This condition contributes to the development of neurodegenerative diseases as AD and, simultaneously, characterizes the cellular environment of the pathology.

AD and aging are characterized by the impairment of proteostasis. Neuronal cells are particularly sensitive to protein misfolding that leads to disrupted function of synapses, apoptosis and selective neuronal death [5]. Furthermore, misfolded proteins lose their physiological activity and acquire neurotoxicity, leading to chronic inflammation [6]. The endoplasmic reticulum (ER) is the largest multifunctional organelle in the cell and the principal site for the biosynthesis of proteins, post-translational modification, folding and assembly of newly synthesized proteins [7]. 

Changes in the ER’s structure, integrity, and function are extremely sensitive. Even when numerous proteins and complexes are devoted to the correct folding of proteins, some proteins do not complete the functional form and are misfolded or aggregated [8]. The development of ER stress is one consequence of the accumulation of misfolded protein. Interestingly, ER stress markers often co-localize with protein aggregates [9].

These cellular responses are the results of an integrated intracellular signaling cascade known as the unfolded protein response (UPR). The UPR is regulated by the activation of three sensor transmembrane proteins: inositol-requiring enzyme 1α (IRE1α), protein kinase RNA-like ER kinase (PERK), and activating transcription factor 6 (ATF-6) [10]. 

Increased levels of UPR activation markers have been observed in the brain tissue of AD patients, as compared to healthy individuals. Indeed, in AD neurons showing abnormally phosphorylated Tau protein, the expression levels of the chaperone BiP, PERK and IRE1α were higher than in control tissue [11]. Remarkably, levels of IRE1α phosphorylation directly correlated with the Braak stage of pathology in patients with AD [12]. 

Often in chronic neurodegenerative diseases, ER stress stimulates autophagic activities; however, failure in clearing the aggregated proteins and the impairment of UPR or autophagy leads to the accumulation of misfolded proteins and the progression of neurodegeneration [13]. Autophagy is an important mechanism, especially for neurons that are unable to discard misfolded proteins [14]. ER stress may regulate autophagy via the PERK/eIF2α/ATF-4 signaling pathway. 

The aim of the present work was to identify potential new biomarkers significantly dysregulated in AD, with particular attention to ER stress that characterizes not only neurodegeneration but also the aging process. 

For this purpose, it has been used an integrated approach of behavioral tests, bioinformatics and biomolecular analysis in a murine model of AD induced by intracerebroventricular (i.c.v.) injection of Aβ_1–42_ oligomers at different ages. The cognitive deficit due to AD and age have been assessed with behavioral tests such as Y-maze and Morris water maze tests. The RNA sequencing analysis allowed identification through bioinformatics software such as gene set enrichment analysis (GSEA), protein analysis through evolutionary relationships (PANTHER), and gene ontology enrichment analysis and visualization (GOrilla), several pathways and molecular functions involved in the cellular response to AD and aging, and then confirmed by Western Blotting.

## 2. Results

The effects of Aβ_1–42_ oligomers in the UPR of young and aged mice were evaluated in a murine model of AD induced by i.c.v. injection of 6 µL of Aβ_1–42_ oligomers in C57BL/6 mice at 3 and 18 months. Animals were divided into four experimental groups: two groups received i.c.v. injection of Aβ_1–42_ oligomers at 3 or 18 months (3Aβ and 18Aβ), and two received the same amount of vehicle solution with the same modalities as the others (3Vh and 18Vh).

### 2.1. Aβ_1–42_ Induced Cognitive Impairment in AD Aged Mice

**Figure 1:**
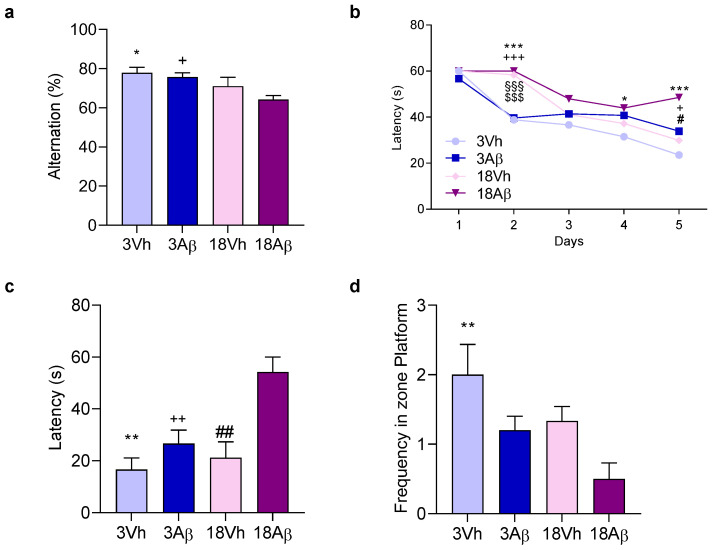
Effects of i.c.v. injection with Aβ_1–42_ oligomers in young and aged mice on the performance in the Y-maze test (**a**) and in the training (**b**) and probe trials (**c**,**d**) of the Morris water maze test (**b**–**d**). In the Y-maze test, the spontaneous alternation percentage was recorded in a 5 min trial. The training trials for the Morris water maze test were carried out for 5 days (four per day); the probe trial was performed on day 6—the escape latency (**c**), and the frequency in the platform zone (**d**) were recorded in the probe test. The values are expressed as mean ± SEM (*n* = 7) ((**a**): * *p* < 0.05 18Aβ vs. 3Vh, ^+^ *p* < 0.05 18Aβ vs. 3Aβ groups; (**b**): day 2 *** *p* < 0.001 18Aβ vs. 3Vh, ^+++^ *p* < 0.001 18Aβ vs. 3Aβ, ^§§§^ *p* < 0.001 18Vh vs. 3Vh, ^$$$^ *p* < 0.001 18Vh vs. 3Aβ; day 4 * *p* < 0.05 18Aβ vs. 3Vh; day 5 *** *p* < 0.001 18Aβ vs. 3Vh, ^+^ *p* < 0.05 18Aβ vs. 3Aβ, ^#^ *p* < 0.05 18Aβ vs. 18Vh; (**c**): ** *p* < 0.01 18Aβ vs. 3Vh, ^++^ *p* < 0.01 18Aβ vs. 3Aβ, and ^##^ *p* < 0.01 18Aβ vs. 18Vh; (**d**): ** *p* < 0.01 vs. 3Vh; mixed model or ordinary one-way ANOVA, post hoc test Tukey).

Firstly, the cognitive impairment was evaluated by behavioral investigation, starting from ten days after the surgery. The Y-maze test (Figure 1a) and the Morris water maze test (Figure 1b–d) were assessed to evaluate the spatial memory and learning impairment during the aging process and as a consequence of the Aβ_1–42_ oligomer injection.

The Y-maze test in the 18Aβ group showed a significant reduction in the alternation percentage as compared to both 3Aβ and 3Vh animals (Figure 1a). In the Morris water maze training, aged mice learned slowly the platform location as shown on day 2, but at the end of the training Aβ mice (3Aβ and 18Aβ) spent more time finding the platform (Figure 1b). During the probe trial, the platform was removed and animals were allowed to swim freely in the pool. The latency for the first entry and the frequency in the platform zone were recorded (Figure 1c,d). Results obtained showed an increase in the latency for the first entry and a decrease in the frequency in the platform zone in 3Aβ and 18Aβ mice, but significantly only in 18Aβ mice. 

### 2.2. Aβ_1–42_ Injection Impaired Proteostasis in AD Aged Mice

At the end of the behavioral assessment, mice were sacrificed, and the hippocampi of both hemispheres were extracted and proceeded for further analysis. In total, 15 samples (4 samples for 3Vh, 3Aβ and 18Aβ, and 3 samples for 18Vh) were selected for RNA extraction and sequencing. The expression data obtained from the sequencing were analyzed and filtered using a *p*-adjusted cutoff of <0.05 and –2 < log2(FC) > 2. Since the number of counts was not high for the 18Vh and 18Aβ groups, in the comparison between these two experimental groups we used a *p*-value < 0.05 as a cutoff. To better understand the role of the aging process in our AD model and how it correlates with Aβ_1–42_ oligomers' neurotoxic activity, we performed the following investigation using three different sets of analysis. In the specific, we compared the 18Aβ group to the 3Vh, 3Aβ, and to 18Vh groups. In this way, it was possible to highlight the role of the aging process (18Aβ vs. 3Aβ), the role of Aβ_1–42_ oligomers in the elderly (18Aβ vs. 18Vh) and both the conditions the aging and the role of Aβ_1–42_ oligomers (18Aβ vs. 3Vh). RNA-seq data were generated and revealed 22,466 for the analysis 18Aβ vs. 3Vh (up 259 and down 2469), 21,769 differentially expressed genes for the analysis 18Aβ vs. 3Aβ (up 176 and down 1913), and 19,600 for the analysis 18Aβ vs. 18Vh (up 90 and down 176). 

**Figure 2:**
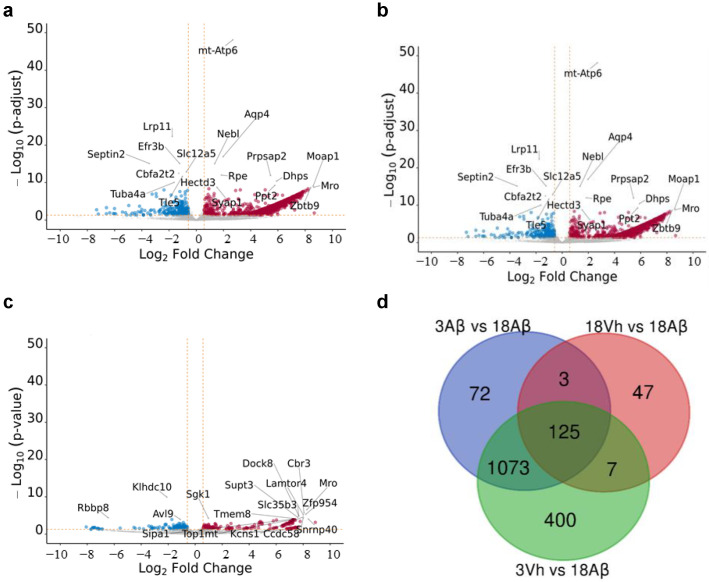
Volcano plot and Venn diagram of significant dysregulated genes in comparison 18Aβ vs. 3Vh (**a**), 18Aβ vs. 3Aβ (**b**), and 18Aβ vs. 18Vh (**c**). The Volcano plot indicates log 10 (*p*-adjusted or *p*-value) for genome-wide genes (Y-axis) plotted against their respective log 2 (fold change) (X-axis). The vertical and horizontal dashed orange lines show the cut-off of fold-change =  ± 1.5, and of *p*-value = 0.05. The red and blue dots represent significantly up- and down-regulated genes, respectively. In the Venn diagram (**d**), the number in each circle represents the amount of differentially expressed genes between the different comparisons. The overlapping number stands for the mutual differentially expressed genes between the different comparisons and the non-overlapping numbers specify the genes unique to each condition.

Indeed, with aging, the samples showed a minor content/quality of RNA that indicates a poor outcome for the proteostasis network. We then used volcano plots to visualize the distribution of significantly dysregulated genes in all comparisons performed (Figure 2a–c).

After that, a Venn diagram among all significantly dysregulated genes was designed, and 125 genes shared by all the comparisons conducted were highlighted (Figure 2d). Interestingly, 18Vh and 18Aβ mice shared only three genes with 3Aβ and 18Aβ mice, as well as seven with 3Vh and 18Aβ. The two comparisons which share the largest number of significantly dysregulated genes are 3Aβ and 18Aβ, and 3Vh and 18Aβ.

**Figure 3:**
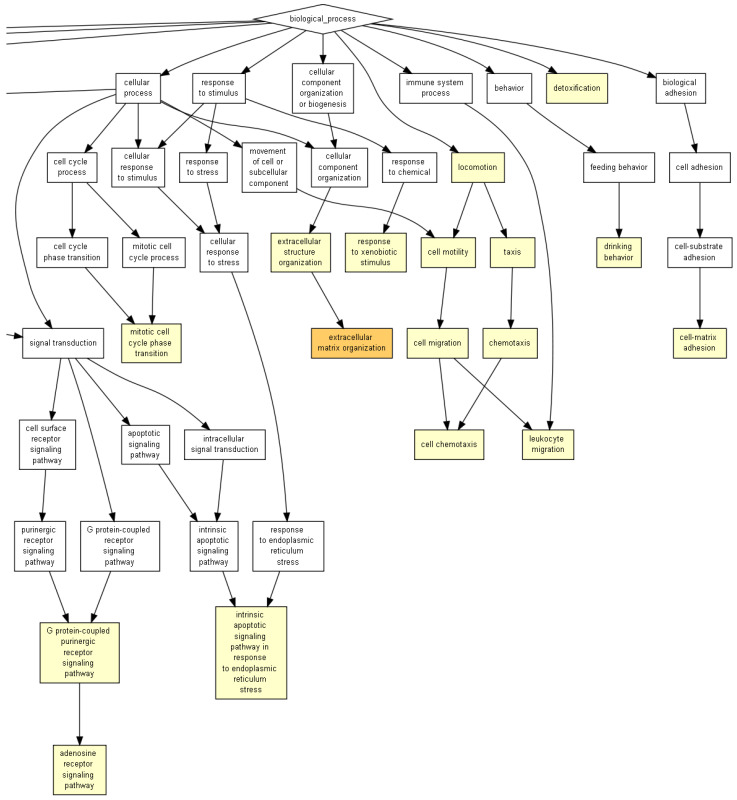
GOrilla reveals the GO terms in the process gene ontology domains, enriched with genes differentially expressed in 18Aβ when compared to 3Vh, the image shows the enlargement of the picture provide as Figure S2 in the Supplementary Material. The pathway analysis was conducted on 1605 differentially expressed genes (*p* < 0.05). Yellow squares represent the GO terms with 10^−3^ < *p* < 10^−5^, and orange squares represent 10^−5^ < *p* < 10^−7^.

**Figure 4:**
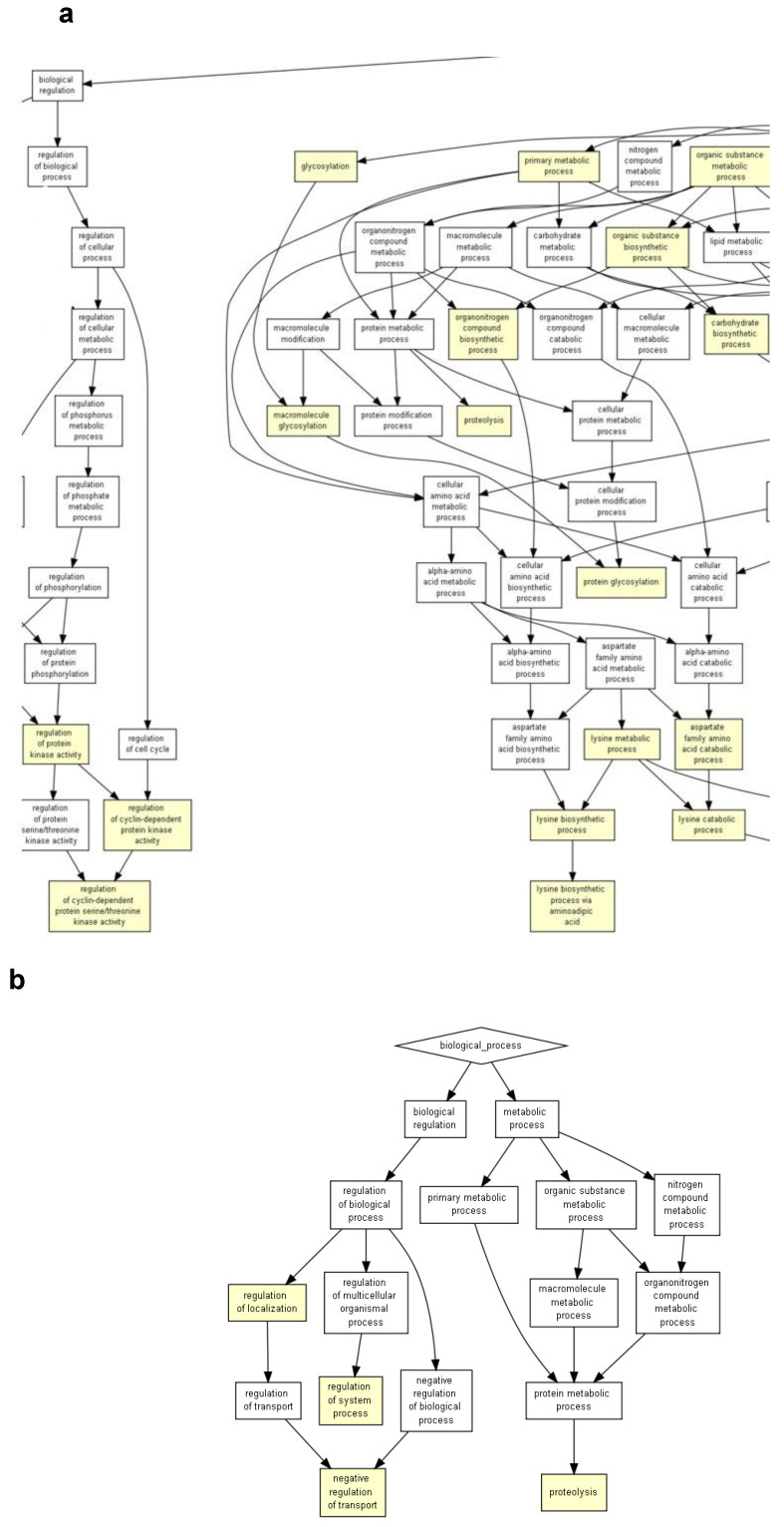
GOrilla reveals the GO terms in the process gene ontology domains, enriched with genes differentially expressed in 18Aβ when compared to 3Aβ, the image shows the magnification of Figure S2 in Supplementary Material 3 (**a**) and in 18Aβ when compared to 18Vh (**b**). The pathway analysis of 18Aβ vs. 3Aβ (**a**) was conducted on 1273 differentially expressed genes, and 18Aβ vs. 18Vh (**b**) was conducted on 182 differentially expressed genes (*p* < 0.05). Yellow squares represent the GO terms with 10^−3^ < *p* < 10^−5^, and orange squares represent 10^−5^ < *p* < 10^−7^.

We conducted a gene ontology (GO) analysis with GOrilla software (https://cbl-gorilla.cs.technion.ac.il/ accessed on 22/12/2022) on the significantly dysregulated genes in all three comparisons. Significantly dysregulated biological processes were shown for the comparison of 18Aβ vs. 3Vh (Figure 3), 18Aβ vs. 3Aβ (Figure 4a), and 18Aβ vs. 18Vh (Figure 4b). The list of significative dysregulated processes is included in the Supplementary Material in Tables S2–S4.

The first analysis highlighted the significant dysregulation of GO terms involved in “regulation protein kinase activity”, “proteolysis”, “detoxication” and “intrinsic apoptotic signaling pathways in response to endoplasmic reticulum stress” (10^−3^ < *p* < 10^−5^), the entire image is shown as Figure S1 in the Supplementary Material. 

The analysis of 18Aβ vs. 3Aβ (Figure 4a, magnification of Figure S2 in Supplementary Material) highlighted the significant dysregulation of GO terms involved in the “regulation protein kinase activity”, “proteolysis”, “protein glycosylation”, “detoxication” (10^−3^ < *p* < 10^−5^), and “cellular process” (10^−5^ < *p* < 10^−7^).

Finally, the analysis conducted on the comparison between 18Vh and 18Aβ (Figure 4b) showed the involvement of GO terms related to “proteolysis” and “regulation of system process” (10^−3^ < *p* < 10^−5^).

We analyzed all the genes involved in the significantly highlighted process obtained by the GOrilla analysis (10^−3^ < *p* < 10^−7^) and we observed that all GO terms showed the involvement of at least 1 gene of the 125 significantly dysregulated among all the comparisons. In total, 68 genes were highlighted in the GO term analysis and summarized in Table S1.

### 2.3. Aβ_1–42_ Injection Impaired UPR and Induced ER Stress in AD Aged Mice

**Figure 5:**
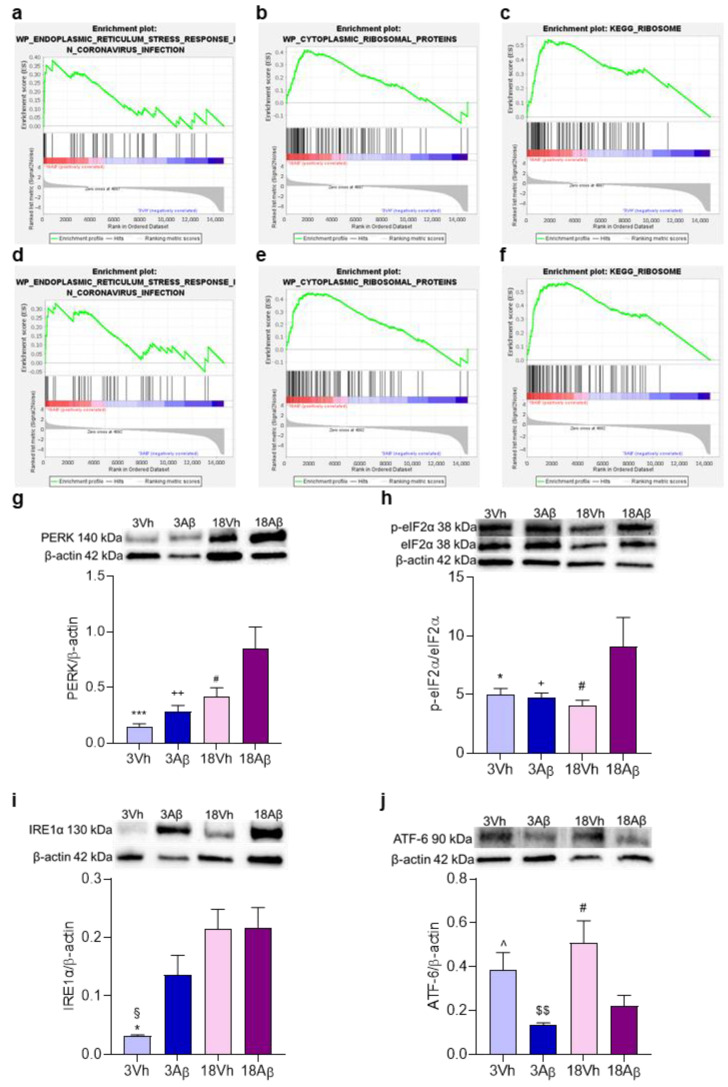
Effects of i.c.v. injection with Aβ_1–42_ oligomers in young and aged mice on UPR. Enrichment plots (**a**–**f**) for core enrichment genes were generated by GSEA using the WP (**a**–**d**) and KEGG (**e**,**f**) gene sets. Comparison identified the enrichment of the “endoplasmic reticulum stress response in coronavirus infection” gene set in 18Aβ vs. 3Vh ((**a**); NES 1.48, FDR 0.93) and in 18Aβ vs. 3Aβ ((**d**); NES 1.35, FDR 1.0); the “cytoplasmic ribosomal proteins” gene set in 18Aβ vs. 3Vh ((**b**); NES 1.22, FDR 1.0) and in 18Aβ vs. 3Aβ ((**e**); NES 1.18, FDR 1.0); the “Ribosome” gene set in 18Aβ vs. 3Vh ((**c**); NES 1.53, FDR 0.28) and in 18Aβ vs. 3Aβ ((**f**); NES 1.41, FDR 0.46). Protein levels of PERK (**g**), p-eIF2α (**h**), IRE1α (**i**), and ATF-6 (**j**) were determined by Western blotting in the hippocampal samples at 140, 38, 130, and 90 kDa, respectively, and using total eIF2α, and β-actin (42 kDa) as loading control. **Top**: representative images of the proteins of interest expressions. **Bottom**: quantitative analysis of the Western blotting results. The graphs show densitometry analysis of the bands appertaining to the protein of interest. The values are expressed as mean ± SEM (*n* = 7) of each group. ((**g**): # *p* < 0.05 18Aβ vs. 18Vh, ^++^ *p* < 0.01 18Aβ vs. 3Aβ, and *** *p* < 0.001 18Aβ vs. 3Vh; (**h**): * *p* < 0.05 18Aβ vs. 3Vh, ^+^ *p* < 0.05 18Aβ vs. 3Aβ, and ^#^ *p* < 0.05 18Aβ vs. 18Vh; (**i**): * *p* < 0.05 18Aβ vs. 3Vh, ^§^ *p* < 0.05 18Vh vs. 3Vh; (**j**): ^ *p* < 0.05 3Aβ vs. 3Vh, ^$$^ *p* < 0.01 18Vh vs. 3Aβ, and ^#^ *p* < 0.05 vs. 18Vh; one-way ANOVA, post hoc test Tukey).

To better understand the involvement of the pathways highlighted by the GO analysis, an enrichment analysis of ER involvement (Figure 5a–f) and expression of protein levels related to UPR and ER stress were assessed (Figure 5g–j). The GSEA analysis showed the enrichment of genes involved in ER stress and activity in the 18Aβ group as compared to the other experimental groups (Figure 5a–f). 

Moreover, the activation of the three pathways involved in the ER stress—PERK/p-eIF2α (Figure 5g,h), IRE1α (Figure 5i), and ATF-6 (Figure 5j)—was significantly up-regulated in 18Aβ mice. In particular, the activation of the PERK/p-eIF2α pathway was significant in 18Aβ mice, while IRE1α showed an increase in 3Aβ mice, which became significant in the 18Vh and 18Aβ groups. Moreover, ATF-6 showed a significant decrease in its activation related to Aβ but not to the age of the mice.

**Figure 6:**
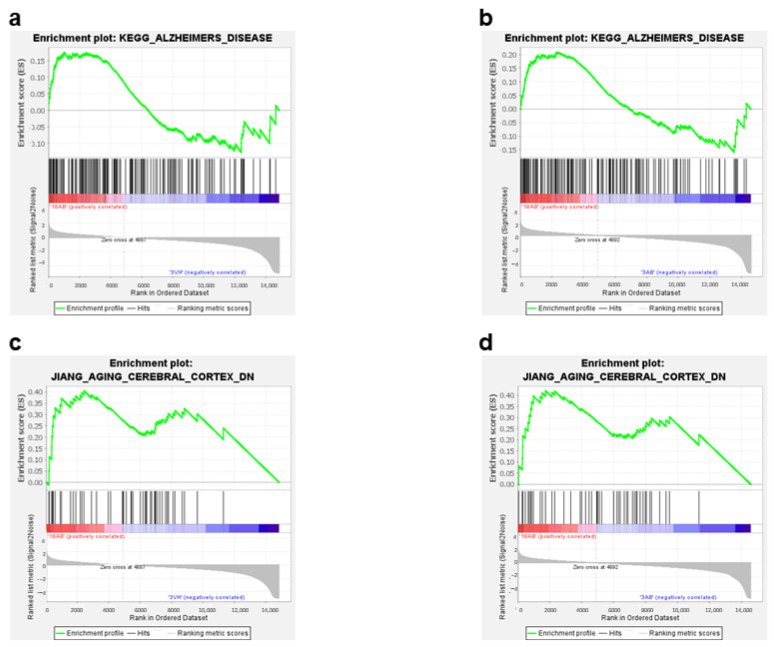
Enrichment plots (**a**–**d**) for core enrichment genes were generated by GSEA using the KEGG (**a**,**b**), and JIANG (**c**,**d**) gene sets. Comparison identified the enrichment of the “Alzheimer’s disease” gene set in 18Aβ vs. 3Vh ((**a**); NES 0.82, FDR 1.0) and in 18Aβ vs. 3Aβ ((**b**); NES 0.89, FDR 0.73); the “Aging cerebral cortex” in 18Aβ vs. 3Vh ((**c**); NES −1.97, FDR 0.06), and in 18Aβ vs. 3Aβ ((**d**); NES 1.62, FDR 1.0).

Furthermore, the GSEA analysis highlighted the enrichment of genes involved in AD (Figure 6a,b) and in the aging process (Figure 6c,d) in the 18Aβ group as compared to young animals of both experimental groups. The same analysis conducted with TOPPGENE highlighted the up-regulation of genes involved in “neurodegenerative disorders” (DisGeNET: C0524851; *p*-value 0.00067; FDR 0.3) in the 18Aβ group as compared to the 3Aβ group.

### 2.4. Aβ_1–42_ Injection Induced Oxidative Stress in AD Aged Mice

**Figure 7:**
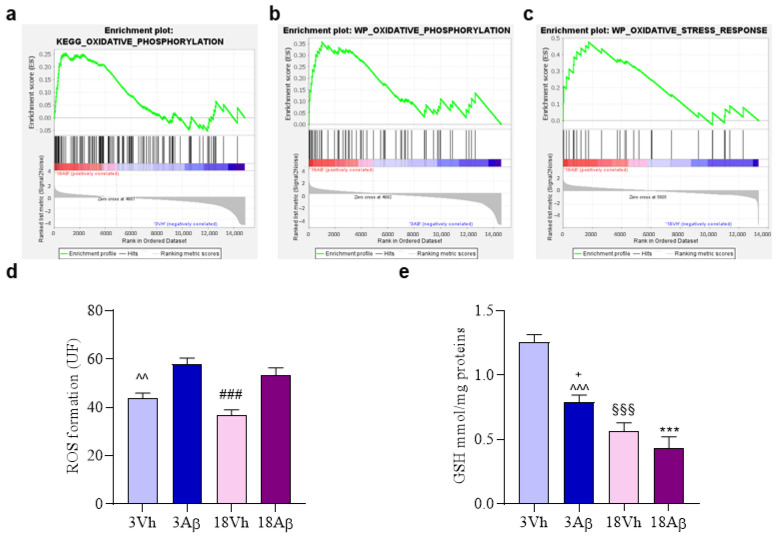
Effects of i.c.v. injection with Aβ_1–42_ oligomers in young and aged mice on cellular redox status. Enrichment plots (**a**–**c**) for core enrichment genes were generated by GSEA using the KEGG (**a**,**b**) and WP (**c**) gene sets. Comparison identified the enrichment of the “Oxidative phosphorylation” gene sets in 18Aβ vs. 3Vh ((**a**); NES 1.09, FDR 0.84) and in 18Aβ vs. 3Aβ ((**b**); NES1.14, FDR 0.63) and the “Oxidative stress response” gene set in 18Aβ vs. 18Vh ((**c**); NES 1.73, FDR 0.51). ROS formation was evaluated in the hippocampal samples based on DCF’s fluorescence emission at 535 nm after excitation at 485 nm. The values are expressed as mean ± SEM (n = 7) of fluorescence intensity arbitrary units (UF) of each experimental group (**d**). GSH content was measured using a colorimetric assay in the hippocampal samples. The values are calculated using a standard calibration curve and expressed as mean ± SEM (*n* = 7) of mmol GSH/mg protein (**e**) ((**d**): ^^^^ *p* < 0.01 3Aβ vs. 3Vh, ^###^ *p* < 0.001 18Aβ vs. 18Vh; (**e**): *** *p* < 0.001 18Aβ vs. 3Vh, ^^^^^ *p* < 0.001 3Aβ vs. 3Vh, ^§§§^ *p* < 0.001 18Vh vs. 3Vh, ^+^ *p* < 0.05 18Aβ vs. 3Aβ; one-way ANOVA, post hoc test Tukey).

GSEA revealed that genes linked to “oxidative phosphorylation” (Figure 7a,b) and “oxidative stress response” (Figure 7c) were enriched in the 18Aβ experimental group as compared to 3Vh (Figure 7a), 3Aβ (Figure 7b), and 18Vh (Figure 7c). Moreover, the cellular redox status was investigated by the evaluation of reactive oxygen species (ROS) formation (Figure 7d) and glutathione (GSH) levels (Figure 7e). Results showed a significant increase in ROS formation after the Aβ_1–42_ oligomer injection in both young and aged animals as compared to the corresponding Vh group. Moreover, GSH levels decreased significantly both with aging and after the Aβ injection.

### 2.5. Aβ_1–42_ Injection Induced the Activation of PI3K/Akt/Gsk3 and MAPK/ERK Pathways in AD Aged Mice

**Figure 8:**
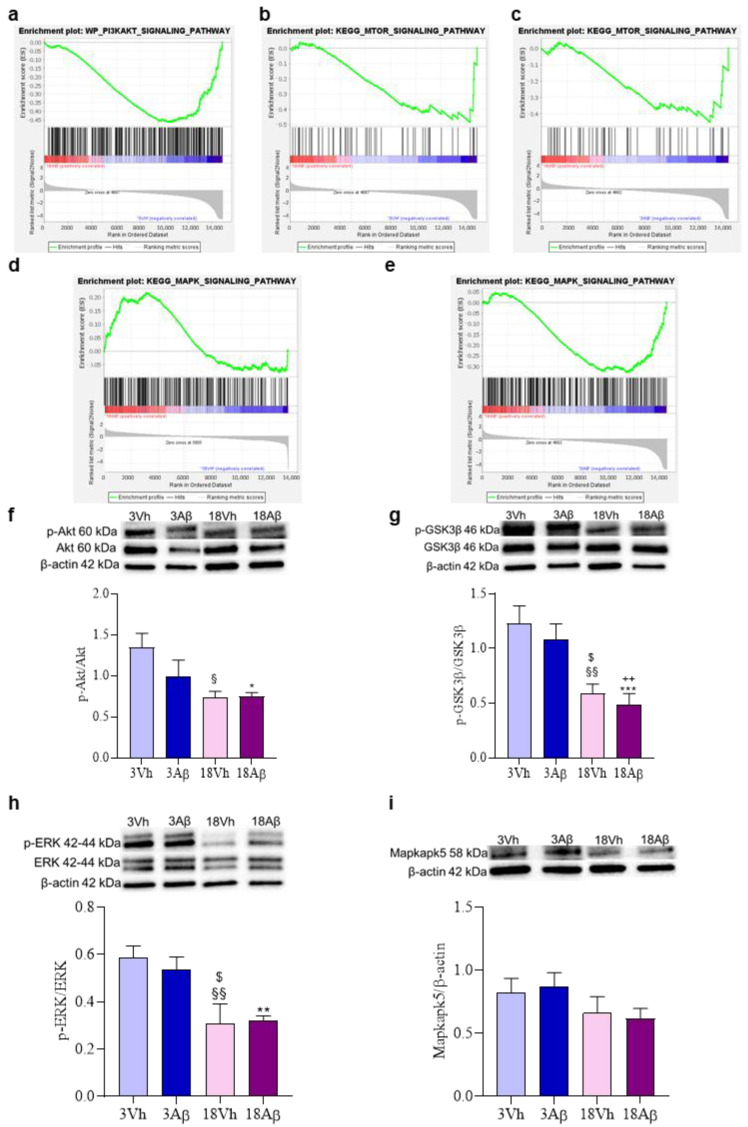
Effects of i.c.v. injection with Aβ_1–42_ oligomers in young and aged mice on MAPK and PI3K/Akt/mTOR signaling pathways. Enrichment plots (**a**–**e**) for core enrichment genes were generated by GSEA using the WP (**a**), KEGG (**b**–**e**) gene sets. Comparison identified the enrichment of the “PI3KAKT signaling pathway” ((**a**); NES −2.08, FDR 0.03) and “MTOR signaling pathway” ((**b**); NES −2.11, FDR 0.03) gene sets in 18Aβ vs. 3Vh, the enrichment plot of the “MTOR signaling pathway” ((**c**); NES −2.11, FDR 0.03), and “MAPK signaling pathway” ((**e**); NES −1.83, FDR 0.03) gene sets in 18Aβ vs. 3Aβ, and “MAPK signaling pathway” ((**d**); NES 1.21, FDR 0.77) gene set in 18Aβ vs. 18Vh. The protein levels of p-Akt (**f**), p-GSK3β (**g**), p-ERK1/2 (**h**), and Mapkapk5 (**i**) were determined by Western blotting in the hippocampal samples at 60, 46, 42/44, and 58 kDa, respectively, and using Akt, GSK3β, ERK1/2 and β-actin (42 kDa) as loading control. Top: representative images of the protein of interest expressions. Bottom: quantitative analysis of the Western blotting results. The graphs show densitometry analysis of the bands appertaining to the protein of interest. The values are expressed as mean ± SEM (*n* = 7) of each group. ((**f**): * *p* < 0.05 18Aβ vs. 3Vh, ^§^ *p* < 0.05 18Vh vs. 3Vh; (**g**): ^§§^ *p* < 0.01 18Vh vs. 3Vh, *** *p* < 0.001 18Aβ vs. 3Vh, ^$^ *p* < 0.05 18Vh vs. 3Aβ, ^++^ *p* < 0.01 18Aβ vs. 3Aβ; (**h**): ^$^ *p* < 0.05 18Vh vs. 3Aβ, ** *p* < 0.01 18Aβ vs. 3Vh, ^§§^ *p* < 0.01 18Vh vs. 3Vh; one-way ANOVA, post hoc test Tukey).

Finally, GSEA analysis highlighted genes linked to the “PI3KAKT signaling pathway” (Figure 8a) or “mTOR signaling pathway” (Figure 8b,c) were not enriched in 18Aβ as compared to the other experimental groups. On the contrary, the enrichment analysis of genes involved in the “MAPK signaling pathway” (Figure 8d,e) showed the enrichment in 18Aβ as compared to 18Vh, but not to 3Aβ. 

Thus, the involvement of the phosphatidylinositide-3-OH kinase (PI3K)/Akt and MAPK/ERK pathways were investigated by the assessment of the phosphorylation of Akt, GSK3β, ERK1/2, and Mapkapk5 (Figure 8f–i). Results obtained showed a significant decrease in the phosphorylation of Akt in aged animals (both 18Vh and 18Aβ; Figure 8f), and GSK3β, which corresponds to a significant increase in the activity of this kinase (Figure 8g). Moreover, a decrease in the phosphorylation of ERK1/2 and Mapkapk5 in the 18Vh and 18Aβ groups as compared to the 3Vh and 3Aβ groups was observed (Figure 8h,i).

## 3. Discussion

AD is a complex multifaced disease that cannot be efficiently treated by modulating a single target but requires multitarget drug treatment to address the different pathological aspects of this devastating disorder. There is an urgent need to find early predictors of the disease, especially for sporadic cases, to have more chances to start early treatments and neuroprotective strategies to give years of high-quality life to patients. The most important risk factor for AD is the aging process and the related involvement of the neuroinflammatory response, the decline in antioxidative resources, and the increasing accumulation of misfolded proteins due to the failure of the UPR. 

In the present study, the role of the aging process was evaluated in a mice model of AD induced by i.c.v. injection of Aβ_1–42_ oligomers in young and aged mice (3 and 18 months). 

The cognitive impairment was first investigated by behavioral assessment using Y-maze and Morris water maze tests. According to Yang et al., results obtained showed a significant impairment after the lesion induced in old mice as compared to younger animals [15]. The older and healthy experimental group did not show a significant alteration to their learning and mnesic capacities as compared to younger mice. These results indicate that Aβ_1–42_ oligomer injection induces a different output according to the age of the animals.

Gene expression and GSEA analysis showed the dysregulation of different pathways in the comparisons performed, highlighting the involvement of the oxidative response, PI3K/Akt/mTOR and ERK pathways, and alterations at ER level. Interestingly, the enrichment analysis of the comparison between 18Aβ and 3Vh (up 259 and down 2469) showed dysregulation of genes involved in pathways related to AD and the aging process. 

The GO terms analysis conducted on GOrilla using significant dysregulated genes confirmed the involvement of ER and the regulation of protein kinase activity. The three comparisons conducted (18Aβ vs. 18Vh, 18Aβ vs. 3Aβ, 18Aβ vs. 3Vh) showed the dysregulation of 125 common genes; among these, 68 were involved in the pathways highlighted by the GO terms analysis. Following the bioinformatic analysis, biomolecular assessment was performed to validate the bioinformatic results. 

The involvement of ER stress and UPR highlighted by the enrichment analysis was then investigated by Western Blotting. Several studies have shown that ER stress is a common pathological feature of AD [12] and mediates, in part, the occurrence of neuronal loss triggered by Aβ [16]. Remarkably, human neurons derived from induced pluripotent stem cells of AD patients revealed that ER stress is a prominent feature of this disease model [17]. Importantly, the well-known effects of UPR signaling on protein translation repression were shown to contribute to the cognitive impairment observed in AD models involving the activation of the stress sensor PERK and the phosphorylation of eIF2α [18]. The UPR is triggered by three main branches: the PERK/p-eIF2α axis, IRE1α, and ATF-6. In resting cells, all three ER stress receptors are in an inactive state, but as unfolded proteins accumulate, they are dissociated leading to their activation and triggering the UPR. In physiological conditions, the UPR is a pro-survival response to reduce the accumulation of unfolded proteins and restore normal ER functioning. However, if protein aggregation is persistent and the stress cannot be resolved, signaling switches from pro-survival to pro-apoptotic response. In our model, we observed increased PERK activation with aging and Aβ_1–42_ oligomer treatment that is significant in the 18Aβ group. Moreover, PERK activation leads to the significant increase in phosphorylation of eIF2α in the same experimental group. These results agree to other studies which have shown that PERK activation and the consequent elevation of p-eIF2α attenuates general translation and promotes the synthesis of ATF-4 and BACE1, resulting in the generation of Aβ with a high ability to form toxic senile plaques in AD brains. Moreover, long-term stress conditions may induce the expression of pro-apoptotic proteins via ATF-4, promoting the apoptotic neuronal. In this view, dysregulated translation and increased levels of p-eIF2α may be a major contributor to structural and functional neuronal loss which result in memory impairment [19].

Under ER stress conditions, the stress transducer IRE1α auto-phosphorylates and homodimerizes activating the most conserved UPR signaling branch, which results in a conformational change in the cytosolic region that activates its RNase domain. IRE1α leads to the expression of XBP1s, a potent transcription factor that transactivates a cluster of UPR-target genes and, finally, provides a negative feedback loop that inhibits PERK activation to terminate UPR [20]. At this point, if the UPR has been successful, the ER returns to normal functioning and the cell survives; however, if the stress persists, it might allow the synthesis of pro-apoptotic proteins. According to Duran-Aniotz et al. [21], who have shown the direct association between IRE1α levels and AD severity in the human brain, our results underlined a progressive increase in protein levels which became significant in both 18Vh and 18Aβ groups. Although during aging IRE1α decreases due to the deterioration of the UPR, the persisting cellular stress, also related to the decreasing GSH levels, probably determines the activation of IRE1α and promotes cell death and inflammation [21].

ATF-6, the third axis of UPR, is a type II transmembrane protein that remains inactive in unstressed cells. In ER stress, ATF-6 is activated and translocated to the nucleus from the cytoplasm to induce the transcription of ER chaperones. Several recent studies have demonstrated that ATF-6 counteracts the accumulation of senile plaques and its inactivation is associated with the progression of AD due to the inability of neurons to survive ER stress [22]. According to these considerations, our results showed a significant decrease in ATF-6 levels in young and aged mice treated with Aβ_1–42_ oligomers, but not in age-matched healthy mice. The ATF-6 is the least studied branch of the UPR in the context of aging. Recent evidence in nematodes suggested that the relationship between the ATF-6 and age is more complex. Burkewitz et al. demonstrated in C. elegans that ATF-6 signaling can show detrimental effects with age under physiological conditions, independently of its canonical UPR role in maintaining proteostasis [23]. Moreover, ATF-6 can reduce early ER stress and protect neurons at multiple levels. However, when the prolonged presence of stimuli impairs ER function, the ATF-6 signaling pathway initiates the ER-mediated apoptotic pathway, inducing the expression of pro-apoptotic molecules such as caspase-12 and CHOP to induce apoptosis [24].

According to Leutner et al., in our model, the aging process did not affect ROS production in hippocampal samples as compared to healthy young mice [25]. On the other hand, the lesion with Aβ_1–42_ oligomers induced a significant increase in ROS production both in young and aged mice, showing the pro-oxidative effects of oligomers. Moreover, several studies have shown that GSH levels decrease after the treatment with Aβ_1–42_ oligomers and during aging [26]. Our results confirm this hypothesis and suggest that the increased susceptibility of the AD brain, both young and old, to oxidative damage may be a consequence of GSH depletion.

Increasing evidence suggests that the PI3K/Akt pathway is involved in ER stress. Interestingly, the activation of Akt has been reported in neurons and glial cells of AD patients' postmortem brains [27]. On the contrary, Hosoi et al. showed the dual regulation of Akt that appears to be activated on short-term exposure to ER stress but downregulated on long-term exposure to ER stress [28]. Similarly, our results showed a significant decrease in Akt activation, through its phosphorylation, in both aged experimental groups. Down-regulation of Akt leads to the up-regulation of GSK3β in the hippocampus of AD patients and it is associated with hyperphosphorylated Tau and NFTs depositions [29]. It has been reported that GSK3β stimulates Aβ production; indeed, its pharmacological inhibition or knockdown reduces the processing of APP to Aβ [30]. Consistently, GSK3β over-activation can increase the activity of PS1 and the expression of BACE1, the occurring increase in Aβ activates GSK3β, which in turn triggers Aβ production, resulting in a positive feedback loop [31]. On the contrary, in mouse models of AD, the inhibition of GSK3β reduces Aβ levels, Tau hyperphosphorylation, and cognitive deficits [32]. Furthermore, GSK3β can activate NF-κB signaling, which is also a protein target for PERK, enhancing inflammation [33]. In our result, the activation of PERK may be responsible for GSK3β significant expression in aged mice; indeed, the phosphorylation on Ser9/21 corresponds to the inhibition of the kinase of interest.

Under ER stress, the UPR acts to restore protein homeostasis also through the activation of ERK, a member of the mitogen-activated protein kinases superfamily of signaling pathways, associated with the regulation of proliferation and differentiation as well as cell survival. ERK has a fundamental role in neuroplasticity and its phosphorylation is induced by the binding of neurotrophins to their specific tyrosine kinase receptors or by the neuronal activity leading to glutamate release and binding to its ionotropic and metabotropic receptors [34]. In AD patients and models, mRNA expression and protein levels of ERK show stage-dependent abnormalities [35]. Although transient ERK activation plays an important role in memory-related processes, its persistent activation can lead to cell death [36]. Therefore, either hyper- or hypoactivation of ERK could contribute to the disorder. Indeed, Webster et al. reported that although in the early phase of AD is possible to observe an extensive activation of ERK, with the progression of the disease, it appears reduced, showing a strong inverse correlation with the Braak stage and the Blessed score for cognition [35]. The functional effect of ERK on dendrites and memory impairment is consistent with the hypothesis that in AD, ERK hypoactivation contributes to cognitive decline. Moreover, several studies have shown that Aβ_25–35_ i.c.v. injection can inhibit ERK phosphorylation [37]. Different Aβ aggregates may have diverse effects; for example, fibrillar Aβ is able to stimulate ERK, while low soluble oligomers initially stimulate but then down-regulate ERK in hippocampal slice cultures [38]. Similarly, in our model, the sustained activation of UPR determines a significant reduction in ERK activation both in healthy and lesioned old mice, also showing the degradation of the neurotrophic activity in aging.

Among the 68 genes significantly dysregulated in all the comparisons investigated, Mapkapk5 has been considered for further investigation. The RNA sequencing analysis showed a progressive decrease in gene expression from healthy young mice to old lesioned experimental group and its decline appears to be related to aging and Aβ_1–42_ treatment. Mapkapk5 is a serine/threonine protein kinase activated through a direct MAPK-dependent pathway to initiate and regulate different cellular processes, such as proliferation, differentiation, apoptosis and gene expression, in response to external stimuli [39]. Improper signaling or dysregulation of the cascades regulated by MAPK enzymes can induce the development or progress of different diseases, such as cancers, diabetes, or developmental disorders. A link between AD and reduced levels of Mapkapk5 has been already proposed [40]. Our results have shown a significant decrease in Mapkapk5 gene expression with the progression of the pathology. Although protein level evaluation did not show a significant decrease, it was possible to observe a trend in this direction. 

## 4. Materials and Methods

### 4.1. Animals

Adult male C57Bl/6 mice (10 weeks old, 25–30 g body weight; Charles River Laboratories Italia Srl, Calco, LC, Italy) were utilized. Mice were housed in a temperature-controlled room (23–24 °C) with free access to food and water and 12 h light/12 h dark cycles. Experimental procedures were carried out from 9:00 a.m. to 3:00 p.m., including control groups in any tests utilized. Briefly, procedures on the mice were completed according to the European Communities Council Directive 2010/63/EU and the current Italian Law on the welfare of laboratory animals (D.Lgs. n.26/2014). The animal protocol was approved by the Italian Ministry of Health (Authorization No. 291/2017-PR) and by the corresponding committee at the University of Bologna. The number of experimental animals was minimized, and care was taken to limit mice suffering. Mice were allowed to acclimate for at least 2 weeks before the beginning of experiments.

### 4.2. Experimental Design

The animals were randomized into four groups (*n* = 7/group): 3Vh, 3Aβ, 18Vh, and 18Aβ. Two groups were treated, one at 3 and one at 18 months, with Aβ_1–42_ oligomers by a unilateral i.c.v. injection, while the others received, at the same age, saline solution (sham groups). After 10 days from the surgery, the mice underwent behavioral assessment. After the behavioral analysis, the animals were deeply anesthetized before being sacrificed by cervical dislocation to collect the hippocampal samples for biomolecular analysis.

### 4.3. Aβ_1–42_ Oligomers Preparation and Injection

Aβ_1–42_ peptides (AnaSpec, Fremont, CA, USA) were solubilized to 1 mg/mL in hexafluoroisopropanol before being sonicated and lyophilized at room temperature. The unaggregated Aβ_1–42_ film obtained was dissolved to a final concentration of 1 mM with sterile dimethylsulfoxide and stored at −20 °C until use. Briefly, to enhance oligomer formation, the Aβ_1–42_ stock was diluted in saline buffer at 40 μM and incubated for 48 h at 4 °C [41].

Animals were anesthetized under gaseous anesthesia (2% isoflurane in 1 L/min O_2_/N_2_O) using an anesthesia system (Ugo Basile, Varese, Italy) and then they were positioned on a mouse stereotaxic frame (myNeuroLab, Leica-Microsystems Co., St. Louis, MO, USA). Anesthesia was maintained with 1.5% isoflurane/O_2_ (1 L/min). The scalp was incised to reveal the skull and identify the bregma to set coordinates. Six microliters of Aβ_1–42_ oligomers (40 μM) were injected i.c.v. using a 10 µL Hamilton syringe at a rate of 0.5 mL/min. After the injection, the needle was left in place for a few minutes before being slowly retracted and the wound was cleaned and sutured (Histoacryl, Aesculap AG, Tuttlingen, Germany). The sham mice received the corresponding volume of saline. The following coordinates were used: anteroposterior: +0.22, mediolateral: +1.0, dorsoventral: −2.5, with a flat skull position. After the surgery mice were collocated under an irradiating lamp to promote recovery.

### 4.4. Y-Maze Test

The spatial working memory was evaluated by recording spontaneous alternation behavior in the Y-maze as described earlier [42]. Briefly, each arm of the maze (Ugo Basile^®^ S.r.L., Gemonio, VA, Italy) was 35 cm long, 15 cm high, and 5 cm wide and converged to a 120° angle. The mice were positioned at the end of one arm and allowed to move freely through the maze for 5 min. The consecutive entry in all three arms was counted as an alternation. Thus, the number of maximum alternations was calculated as the total number of arm entries minus two and the percentage of alternation was calculated as (actual alternations/maximum alternations) × 100 [43].

### 4.5. Morris Water Maze Test

The test was performed using a circular plastic tank (1.0 m diameter, 50 cm height) filled with water and milk (22 °C). The maze was located in a room containing several simple visual, extra-maze cues that were constant throughout the study. A transparent platform was set inside the tank and submerged 1.5 cm below the water surface in the center of one of the four quadrants of the maze [44]. 

A camera was placed to register mice’s movements and send data to an automated tracking system (EthoVision, Noldus, Wageningen, The Netherlands). For each training trial, animals were placed into the pool at one of the four positions selected randomly, and the latency to find the hidden platform was recorded. The platform was located in a constant position throughout the test period in the middle of one quadrant. Mice that could not reach the platform within 60 s were guided to it by the experimenter and allowed to remain there for 10 s. After the trial, each mouse was placed under a warming lamp in a holding cage for 25 s until the next trial. Training trials were conducted four times a day for 5 days. On day 6, the platform was removed, and animals were allowed to swim freely for 60 s. During the probe trial, the escape latency and the frequency in the platform zone were recorded.

### 4.6. Tissue Preparation for Neurochemical Analysis

At the end of behavioral tests, the mice were deeply anesthetized and sacrificed by cervical dislocation. The brains were quickly removed, and the hippocampi of each hemisphere were isolated on ice and transferred to liquid nitrogen. For the protein extraction, the tissues were homogenized in lysis buffer (50 mM Tris, pH 7.5, 0.4% NP-40, 10% glycerol, 150 mM NaCl, 10 μg/mL aprotinin, 20 μg/mL leupeptin, 10 mM EDTA, 1 mM sodium orthovanadate, 100 mM sodium fluoride) and the cytoplasmic protein concentration was determined by the Bradford method (Bio-Rad Laboratories S.r.L., Hercules, CA, USA). 

Total RNA was isolated from the hippocampus using the RNeasy Plus mini kit (Qiagen S.r.L., Milan, Italy). Briefly, the samples were lysed on ice with 1% β-mercaptoethanol by using a homogenizer SHM1 (Stuart, Bibby Scientific Ltd., Staffordshire, UK). The samples were then added to an equal volume of 70% ethanol. The solution was filtered using a cartridge containing a clear silica-based membrane to which the RNA binds. RNA was finally eluted with RNase-free water and stored at −80 °C. 

RNA integrity was evaluated with an Agilent RNA 6000 nano kit using the Agilent 2100 Bioanalyzer System (Agilent Technologies Italia S.p.A., Cernusco sul Naviglio, MI, Italy) by spectrophotometric analysis. Libraries were then prepared using the Qiaseq Stranded total RNA library (Qiagen) and their quantification was assessed with the High Sensitivity DNA assay (Agilent) using the Agilent 2100 Bioanalyzer System (Agilent), and again with the Qubit 1× ds DNA HS kit using Qubit (Thermo Fisher Scientific, Waltham, MA, USA). Finally, two pools were prepared with each sample at the concentration of 2000 pM for the library sequencing.

### 4.7. RNA-Seq Analysis

RNA sequencing was carried out according to the Illumina pipeline on a NextSeq 500 Instrument (Illumina, San Diego, CA, USA) using the NextSeq High Output kit v2 (150 cycles) (Illumina). Obtained sequences were mapped to the mouse genome (GRCm38) using the algorithm HISAT2 [45] and a pre-built genome index downloadable from the HISAT2 website. Then, StringTie [46] was used to assemble and quantify the transcripts in each sample. Finally, expressed transcripts have been normalized using the DeSeq2 package for R. Differential gene expression and downstream analyses were performed using DEseq2 and custom R scripts [47]. For the analysis, gene differential expression tests between 3Vh, 3Aβ, 18Vh and 18Aβ groups were performed for differently aged and treated and not treated mice (3 months treated/not treated, and 18 months treated/not treated) by their log2 fold change (log2FC) from the basal state. As cutoff criteria, we used at least one comparison *p*-value of < 0.05 and −2 < log2(FC) > 2.

### 4.8. Bioinformatic Analysis

Gene expression analysis was performed using the Broad gene set enrichment analysis GSEA tool [48] and the TOPPGENE tool [49]. The C2-curated genes were used as background gene sets using the MsigDB database (http://software.broadinstitute.org/gsea/msigdb/, version 7.4 accessed on 27/07/2022). The screening of up-regulated and down-regulated gene sets for each analysis (3Vh vs. 18Aβ, 3Aβ vs. 18Aβ, and 18Vh vs. 18Aβ) was performed using GSEA enrichment analysis, using parameters recommended for expression datasets with less than 7 replicates for samples were as follows: numbers of permutations (10,000), collapsed datasets due to gene symbols (true), enrichment statistic (classic), metrics for gene ranking (Signal2Noise), gene list sorting model (real), gene list ordering mode (descending), max size (500), and min size (15). The gene ontology (GO) terms were evaluated with the broad tool GOrilla [50].

### 4.9. Determination of Cellular Redox Status

The redox status, in terms of ROS formation, was evaluated by measuring the oxidation of DCFH-DA to 2′7′-dichlorofluorescein (DCF) [51]. The samples (60 μL) were incubated for 30 min with 2 mg/mL of DCFH-DA, to allow the DCFH-DA to be incorporated into any membrane-bound vesicles and the diacetate group to be cleaved by esterases. At the term of incubation, the conversion into the fluorescent product DCF was measured (excitation at 485 nm, emission at 535 nm) using a microplate reader (GENios, TECAN^®^, Männedorf, Switzerland). Background fluorescence (conversion of DCFH-DA in the absence of homogenate) was corrected by the inclusion of parallel blanks. The values were normalized to protein content and expressed as the mean ± SEM of fluorescence intensity arbitrary units (UF) of each experimental group.

### 4.10. Determination of Glutathione Content

GSH content was assessed on samples (50 µL) precipitated with 100 μL of sulfosalicylic acid (4%). The samples were kept at 4 °C for at least 1 h and then subjected to centrifugation at 3000 rpm for 10 min at 4 °C. A volume of 25 μL of the assay mixture and 50 μL of 5-5′-dithio-bis (2-nitrobenzoic acid) (4 mg/mL in phosphate buffer, 0.1 M, pH 7.4) was made up to a total volume of 500 μL. The yellow color that developed was read immediately at 412 nm (GENios, TECAN^®^) and the results were calculated using a standard calibration curve. The values were normalized to protein content and expressed as the mean ± SEM of GSH mmol/mg protein of each experimental group.

### 4.11. Western Blotting

Samples (30 μg proteins) were added to the Laemmli Sample Buffer 4× (Bio-Rad Laboratories S.r.L.) and then separated on 4–15% TGX polyacrylamide gels (Bio-Rad Laboratories S.r.L.) in running buffer (25 mM Tris; 192 mM Glycine; 0,1% (*w*/*v*) SDS pH 8,3) for 30 min at 200 V. Before being electroblotted onto 0.45 μm nitrocellulose membranes in blotting buffer (25 mM Tris; 192 mM Glycine; 20% (*v*/*v*) MeOH pH 8,3) for 1 h at 100 V, gels were photoactivated allowing the immediate visualization of proteins. After the transfer, the total protein signal was visualized by UV excitation to obtain normalization data for the next analysis, and membranes were incubated for 2 h in block solution (5% no-fat powder milk; TBS; 0.05% Tween 20). The membranes were incubated at 4 °C overnight with primary antibody recognizing phospho-GSK3α/β (Ser21/9), phospho-p44/42 MAPK (ERK1/2, Thr202/Tyr204), phospho-eIF2α, PERK, ATF-6, phospho-Akt, IRE1α (1:1000; Cell Signaling Technology Inc., Danvers, MA, USA), or Mapkapk5 (1:1000; EpigenTek, Farmingdale, NY, USA). The next day, after washing with TBS-T (TBS + 0.05% Tween20), the membranes were incubated with a horseradish peroxidase (POD) linked anti-rabbit or anti-mouse secondary antibody (1:5000; Jackson ImmunoResearch Europe Ltd., Ely, Cambridgeshire, UK) for 1 h at room temperature (RT). Immunoreactive bands were visualized by enhanced chemiluminescence (ECL; Bio-Rad Laboratories S.r.L.). The same membranes were stripped (100 mM β-mercaptoethanol; 2% SDS; 62,5 mM Tris pH 6.7) and reprobed with GSK3α/β, p44-42 MAPK, Akt, eIF2α (1:1000; Cell Signaling Technology Inc.), or anti-β-actin (1:1000; Sigma-Aldrich, St Louis, MO, USA) at 4 °C overnight. The data were analyzed by densitometry, using Image Lab 6.1 (Bio-Rad Laboratories S.r.L.). The values were normalized and expressed as the mean ± SEM of the densitometry in each experimental group.

### 4.12. Statistical Analysis

The data were analyzed with the PRISM 9.4.1. software (GraphPad Software, La Jolla, CA, USA) and expressed as mean ± SEM of each experimental group. The difference between the groups was analyzed by mixed model or ordinary one-way ANOVA with Tukey’s post hoc test. The results were considered statistically significant when the *p*-value was less than 0.05.

## 5. Conclusions

In conclusion, the results presented in this study underlined the efficacy of our mouse model, which has shown the ability to produce cognitive dysfunction in a few weeks and allow the identification of a target, Mapkapk5, which is significantly dysregulated in the comparisons performed. The functionality of the ER system seems to be at the center of the apoptotic trigger through chronic stimulation of the UPR pathway. While not a classical target, the many protein/gene factors that control the intrinsic ER stress response system may allow for the creation of highly synergistic AD pharmacotherapeutics. By pharmacologically supplementing the ER stress response system, it is possible to ameliorate AD-related pathological systems that include calcium overload, protein misfolding, amyloidogenic APP processing, microtubular disruption, and ROS protection. As such, a proposed agent would, in theory, act simultaneously in multiple cell processes; even poorly efficacious or bioavailable agents (or series of agents) would have their acute actions amplified by the UPR system. In conclusion, the study provides a better understanding of AD’s pathophysiology and UPR involvement. Further studies need to be addressed to better understand how new therapeutic approaches able to enhance Mapkapk5 levels may interfere with and modulate AD evolution.

## Data Availability

All data generated or analyzed during this study are included in this published article.

## Supplementary Materials

The following supporting information can be downloaded at: https://www.mdpi.com/article/10.3390/ijms242216200/s1.
