# Supplementary material for: The Unfolded Protein Response in a Murine Model of Alzheimer’s Disease: Looking for Predictors
Source: Int J Mol Sci. 2023 Nov 11;24(22):16200. doi: 10.3390/ijms242216200 (PMC10671834; doi:10.3390/ijms242216200)
Supplement: Supplementary file 1 [file ijms-24-16200-s001.zip › ijms-2657383-supplementary.pdf]

Table S1. Significantly dysregulated genes in GOrilla analysis and common to all comparisons

Abcg2	Chkb	Eme2	Haghl	Mapkapk5	Nudcd2	Polr3b
Ahr	Chrd	Exosc4	Hfe	Mboat1	Nup85	Prpsap2
Alkbh7	Csgalnact2	Fancg	Id1	Mcm7	Parp9	Psmg2
Amn1	Dcxr	Fcgr1	Ifi203	Moap1	Pcdh20	Ptprc
Ap5z1	Dhodh	Fdx1	Igtp	Mospd3	Pcdha2	Ptprr
Arhgap25	Dhps	Fn3k	Kcns1	Myd88	Pex10	Rad18
Arghap27	Dock2	Fuz	Kif11	Ncf2	Pigh	Rbm11
B4galt1	Dock8	Gp1bb	Lamtor4	Ndufaf2	Pigx	Sardh
Cbr3	Dph2	Gstm4	Lbp	Nostrin	Plcd3	
Ccnf	Ehbp1l1	Hacl1	Lsm7	Nphp1	Plk5	

Table S2. Significant dysregulated biological process in Figure 3 (18Aβ vs. 3Vh).

Yellow box 10-3 < p < 10-5	Detoxification*
	Locomotion*
	Primary metabolic process
	Secondary metabolic process
	Cellular metabolic process
	Extracellular structure organization*
	Response to xenobiotic stimulus*
	Cell motility*
	Taxis*
	Drinking behavior*
	Regulation of cell adhesion
	Regulation of blood pressure
	Regulation of catalytic activity
	Lipid metabolic process
	Small molecule catabolic process
	Cell-matrix adhesion*
	Chemotaxis*
	Cell migration*
	Mitotic cell cycle phase transition*
	Regulation of systemic arterial blood pressure
	Carbohydrate derivative metabolic process
	Purine-containing compound metabolic process
	Thioester metabolic process
	Organic acid catabolic process
	Leukocyte migration*
	Cell chemotaxis*
	Positive regulation of hydrolase activity
	Regulation of systemic arterial blood pressure mediated by a chemical signal
	Endocrine process
	Proteolysis
	Positive regulation of amine transport
	Regulation of tube size
	Vascular process in circulatory system
	Regulation of systemic arterial blood pressure by hormone
	Organonitrogen compound biosynthetic process
	Carboxylic acid catabolic process
	Intrinsic apoptotic signaling pathway in response to endoplasmic reticulum stress*
	G protein-coupled purinergic receptor signaling pathway*
	Regulation of tube diameter
	Regulation of blood vessel size
	Regulation of systemic arterial blood pressure by renin-angiotensin
	Nucleoside bisphosphate metabolic process
	Monocarboxylic acid catabolic process
	Adenosine receptor signaling pathway*
	Regulation of norepinephrine secretion
	Regulation of kinase activity
	Regulation of blood vessel diameter

	Regulation of systemic arterial blood pressure by circulatory renin-angiotensin
	Regulation of blood volume by renin-angiotensin
	Purine nucleoside bisphosphate metabolic process
	Dopamine biosynthetic process
	Fatty acid catabolic process
	Regulation of protein kinase activity
	Positive regulation of blood vessel diameter
	Brain renin-angiotensin system
	Ribonucleoside bisphosphate metabolic process
	Regulation of mineralocorticoid secretion
	Regulation of cyclin-dependent protein kinase activity
	Regulation of protein serine/threonine kinase acitvity
	Vasodilation
	Acyl-CoA metabolic process
	Lysine biosynthetic process
	Regulation of aldosterone secretion
	Regulation of cyclin-dependent protein serine/threonine kinase activity
	Renal system process involved in regulation of systemic arterial blood pressure
	Lysine biosynthetic process via aminoadipic acid
	Renin-angiotensin regulation of aldosterone production
Orange box 10-5 < p < 10-7	Metabolic process
	Organic substance metabolic process
	Small molecule metabolic process
	Organic acid metabolic process
	Extracellular matrix organization*
	Cellular lipid metabolic process
	Oxoacid metabolic process
	Carboxylic acid metabolic process
	Monocarboxylic acid metabolic process
	Fatty acid metabolic process

***biological processes highlighted in the green box**

Table S3. Significant dysregulated biological process in Figure 4a (18Aβ vs. 3Aβ).

Yellow box 10-3 < p < 10-5	Detoxification
	Locomotion
	Metabolic process
	Biological adhesion
	Movement of cell or subcellular component
	Biosynthetic process
	Organic substance metabolic process*
	Primary metabolic process*
	Glycosylation*
	Extracellular matrix organization
	Cellular biosynthetic process
	Small molecule catabolic process
	Organic acid metabolic process
	Organic substance biosynthetic process*
	Extracellular matrix organization
	Mitotic cell cycle phase transition
	Organic acid catabolic process
	Cellular lipid metabolic process
	Oxoacid metabolic process
	Carbohydrate biosynthetic process*
	Organonitrogen compound biosynthetic process*
	Cellular carbohydrate biosynthetic process
	Carboxylic acid metabolic process
	Proteolysis *
	Macromolecule glycosylation*
	Monocarboxylic acid metabolic process
	Carboxylic acid catabolic process
	Fatty acid metabolic process
	Monocarboxylic acid catabolic process
	Protein glycosylation*
	Fatty acid catabolic process
	Aspartate family amino acid catabolic process*
	Lysine metabolic process*
	Regulation of protein kinase activity*
	L-lysine metabolic process
	Lysine catabolic process*
	Lysine biosynthetic process*
	Regulation of cyclin-dependent protein kinase activity*
	L-lysine catabolic process
	Lysine biosynthetic process via aminoadipic acid*
	Regulation of cyclin-dependent protein serine/threonine kinase activity*
Orange box 10-5 < p < 10-7	Cellular process
	Small molecule metabolic process

***biological processes highlighted in the green box**

Table S4. Significant dysregulated biological process in Figure 4b (18Aβ vs. 18Vh).

Yellow box 10-3 < p < 10-5	Regulation of localization
	Regulation of system process
	Negative regulation of transport
	Proteolysis

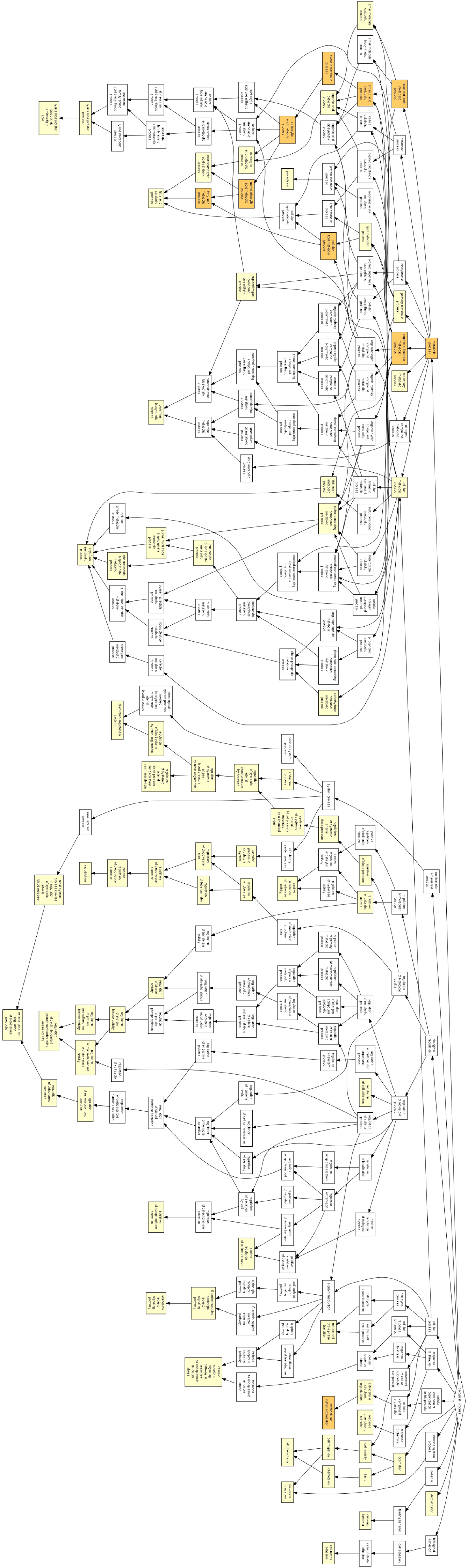

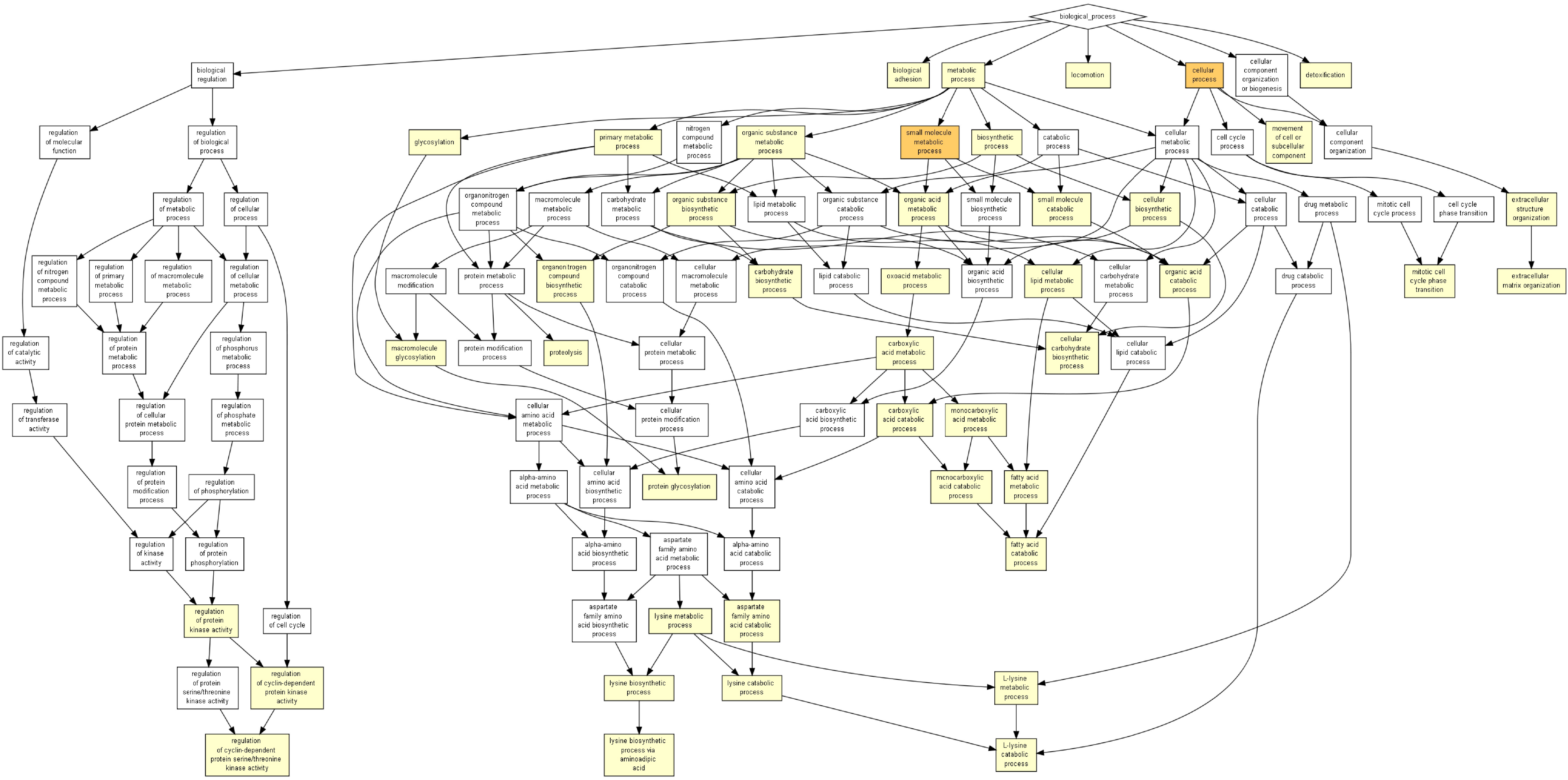
biological_process
biological regulation
biological adhesion
metabolic process
locomotion
cellular process
cellular component organization or biogenesis
detoxification
regulation of molecular function
regulation of biological process
glycosylation
primary metabolic process
nitrogen compound metabolic process
organic substance metabolic process
small molecule metabolic process
biosynthetic process
catabolic process
cellular metabolic process
cell cycle process
movement of cell or subcellular component
cellular component organization
regulation of metabolic process
regulation of cellular process
organonitrogen compound metabolic process
macromolecule metabolic process
carbohydrate metabolic process
organic substance biosynthetic process
lipid metabolic process
organic substance catabolic process
organic acid metabolic process
small molecule biosynthetic process
small molecule catabolic process
cellular biosynthetic process
cellular catabolic process
drug metabolic process
mitotic cell cycle process
cell cycle phase transition
extracellular structure organization
regulation of nitrogen compound metabolic process
regulation of primary metabolic process
regulation of macromolecule metabolic process
regulation of cellular metabolic process
macromolecule modification
protein metabolic process
organonitrogen compound biosynthetic process
organonitrogen compound catabolic process
cellular macromolecule metabolic process
carbohydrate biosynthetic process
lipid catabolic process
oxoacid metabolic process
organic acid biosynthetic process
cellular lipid metabolic process
cellular carbohydrate metabolic process
organic acid catabolic process
drug catabolic process
mitotic cell cycle phase transition
extracellular matrix organization
regulation of catalytic activity
regulation of protein metabolic process
regulation of phosphorus metabolic process
macromolecule glycosylation
protein modification process
proteolysis
cellular protein metabolic process
carboxylic acid metabolic process
cellular carbohydrate biosynthetic process
cellular lipid catabolic process
regulation of transferase activity
regulation of cellular protein metabolic process
regulation of phosphate metabolic process
cellular amino acid metabolic process
cellular protein modification process
carboxylic acid biosynthetic process
carboxylic acid catabolic process
monocarboxylic acid metabolic process
regulation of protein modification process
regulation of phosphorylation
alpha-amino acid metabolic process
cellular amino acid biosynthetic process
protein glycosylation
cellular amino acid catabolic process
monocarboxylic acid catabolic process
fatty acid metabolic process
regulation of kinase activity
regulation of protein phosphorylation
alpha-amino acid biosynthetic process
aspartate family amino acid metabolic process
alpha-amino acid catabolic process
fatty acid catabolic process
regulation of protein kinase activity
regulation of cell cycle
aspartate family amino acid biosynthetic process
lysine metabolic process
aspartate family amino acid catabolic process
regulation of protein serine/threonine kinase activity
regulation of cyclin-dependent protein kinase activity
lysine biosynthetic process
lysine catabolic process
L-lysine metabolic process
regulation of cyclin-dependent protein serine/threonine kinase activity
lysine biosynthetic process via aminoadipic acid
L-lysine catabolic process
